# Pavement Cracks Segmentation Algorithm Based on Conditional Generative Adversarial Network

**DOI:** 10.3390/s22218478

**Published:** 2022-11-03

**Authors:** Jie Kang, Shujie Feng

**Affiliations:** School of Electrical and Control Engineering, Shaanxi University of Science and Technology, Xi’an 710021, China

**Keywords:** pavement cracks segmentation, conditional generative adversarial networks, U-net3+

## Abstract

In long-term use, cracks will show up on the road, delivering monetary losses and security hazards. However, the road surface with a complex background has various disturbances, so it is challenging to segment the cracks accurately. Therefore, we propose a pavement cracks segmentation method based on a conditional generative adversarial network in this paper. U-net3+ with the attention module is used in the generator to generate segmented images for pavement cracks. The attention module highlights crack features and suppresses noise features from two dimensions of channel and space, then fuses the features generated by these two dimensions to obtain more complementary crack features. The original image is stitched with the manual annotation of cracks and the generated segmented image as the input of the discriminator. The PatchGAN method is used in the discriminator. Moreover, we propose a weighted hybrid loss function to improve the segmentation accuracy by exploiting the difference between the generated and annotated images. Through alternating gaming training of the generator and the discriminator, the segmentation image of cracks generated by the generator is very close to the actual segmentation image, thus achieving the effect of crack detection. Our experimental results using the Crack500 datasets show that the proposed method can eliminate various disturbances and achieve superior performance in pavement crack detection with complex backgrounds.

## 1. Introduction

The road is one of the main modes of modern transportation and plays an important role in a country’s economic development. In long-term use, the pavement is prone to damage such as cracks due to vehicle crushing or natural disasters, which can reduce driving comfort and safety and shorten the lifespan of the road. Pavement cracks prevention is an important part of road maintenance. Previous road crack detection methods are mainly traditional manual detection, which require a lot of human and material resources. They are easily affected by the subjective factors of the inspectors, resulting in low detection efficiency. Therefore, the automation of crack detection has high research value and application prospects.

With the development of computer vision, target detection methods based on digital image processing are applied to pavement cracks detection. Commonly used methods can be divided into threshold-based segmentation methods, edge-based segmentation methods, and region-growing-based segmentation methods. The threshold-based segmentation method is to set an appropriate threshold for division according to the grayscale difference between the crack area and the pavement, and the target area is the one that meets the threshold requirements. Akagic et al. [1] implemented threshold segmentation of pavement cracks using a grayscale histogram and OTSU thresholding. Quan et al. [2] improved the segmentation effect of the OTSU algorithm for crack images whose gray histogram did not have obvious bimodal characteristics by adding a probability weighting factor to the gray histogram. The edge-based segmentation methods aim to segment according to the grayscale steps of the crack edge by using the edge detection operators such as the Sobel operator, Prewitt operator, Canny operator, and other operators [3]. Jiang et al. [4] proposed a defect detection algorithm based on the Sobel operator and patch statistics, which is applied to a simple background and has a limited ability to resist background noise. Region-growing-based segmentation methods detect cracks by grouping pixels with similar features into a region. Zhou et al. [5] selected crack seeds by grid cell analysis and used the Euclidean minimum spanning tree to connect them. The experiments proved that the proposed method could effectively detect cracks. Song et al. [6] proposed an improved directional region growing algorithm to detect cracks. If pavement cracks have good continuity and high contrast, digital image processing can achieve good detection results. However, the collected cracks are generally irregular slender black areas, and the continuity will be affected by the texture of the road itself. Road shadows, stains, and others will cause background noise and affect the detection effect.

In recent years, as deep learning does not require manual feature extraction and has better generalization performance and good anti-noise ability, it has been gradually applied in crack segmentation. The Fully Convolutional Network (FCN) transforms the initial network for image classification into the network for image segmentation. Dung et al. [7] proposed a crack detection method based on FCN. This method used the VGG16 network that removes the fully connected layer for encoding the input image to obtain high-level semantic features. For obtaining the image segmentation result, the obtained high-level features were gradually restored to the input image resolution by deconvolution operation and upsampling. Sobel et al. [8] and Park et al. [9] also used various FCNs to detect road cracks. However, when performing segmentation, the FCN does not fully consider the relationship between pixels, which leads to a lack of spatial consistency. Thus, it is not sensitive enough to the details in the image, resulting in inaccurate segmentation results.

This problem can be improved by utilizing feature fusion. Yang et al. [10] proposed a road crack detection algorithm based on the Feature Pyramid and Hierarchical Boosting Network. The network integrated semantic information into the underlying features, then adopted a feature pyramid approach for crack detection. Liu et al. [11] proposed a deep hierarchical convolutional neural network (CNN), which consists of the extended fully convolutional neural network and the deeply supervised nets. The method aggregated multi-scale and multi-level features from the low convolutional layers to the high-level convolutional layers. However, the resolution of the feature map generated by the above methods will continue to decrease after pooling, which results in the loss of spatial location information of cracks and affects the segmentation results.

The encoder–decoder structure can solve the problem of the loss of spatial location information well. Namely, the encoder extracts the location information and image features of cracks through operations such as convolution and pooling. The decoder parses them through operations such as deconvolution and upsampling to restore the spatial dimension of the image and the position information of the pixels. Jenkins et al. [12] proposed a road crack semantic segmentation algorithm based on U-net, which realized the effective segmentation of pavement cracks at the pixel-level. König et al. [13] proposed a fully convolutional U-net architecture that embedded an attention-based gating mechanism and residual connections to preserve only the spatially correlated features of the feature map in skip connections. Gao et al. [14] proposed a crack segmentation method for asphalt pavement images based on the Generative Adversarial Network (GAN) and fused the image segmentation methods CU-net and FU-net with GAN. The precision and recall exceeded the original method by 10%~20%, indicating that adding a GAN to the original segmentation method has better model generalization and robustness.

Although the above methods have achieved good results in pavement crack detection, there are still some problems to be solved, such as the inaccuracy of segmentation results caused by various noises in the detection process. To overcome these problems and improve the accuracy of segmentation, this paper addresses this challenge from three perspectives. First, we combined the improved U-net3+ network with the conditional generative adversarial network (CGAN) and proposed a road crack detection method based on the CGAN. The introduction of GAN in semantic segmentation tasks can enhance spatial long-distance dependence. GAN can evaluate the joint distribution of labels at each pixel position as a whole. Compared with non-adversarial training methods, it pays more attention to the high-order spatial consistency of data and can obtain more accurate and smooth segmentation results. Second, we used the U-net3+ network with the attention module as the generator of the CGAN. To solve the problem of noise interference in road images, we proposed a parallel attention mechanism, which redistributes the weight of crack features from the two dimensions of channel and space, and then fuses the redistributed features to obtain more complementary crack features and suppress the interference. Third, we used a weighted hybrid loss function to improve the accuracy of segmented images. If the segmentation performance is entirely dependent on adversarial losses, then the output segmentation mask may not always have the same global structure as the manual annotation. Therefore, the cross-entropy loss between the generated segmentation mask and the manual segmentation mask was added to the loss function of the generator to improve the segmentation performance by penalizing the distance between them.

To summarize, the contributions of this paper are threefold:

(1) U-net3+ is used as the generator of CGAN, and PatchGAN is used as the discriminator of CGAN for pavement cracks segmentation.
(2) The parallel attention mechanism is embedded into the generator to highlight crack features and suppress noise features.
(3) A weighted hybrid loss function is proposed to improve the segmentation accuracy of cracks.
(4) This paper is organized as follows. Section 2 presents related work on pavement crack segmentation. Section 2 presents preliminary work about U-net3+ and CGAN. Section 4 introduces the design of our proposed network generator, discriminator, and loss function. Section 5 describes the dataset, evaluation metrics, and experimental content used in the experiments. Section 6 concludes the paper.

## 2. Related Work

At present, some non-destructive testing techniques have made some progress in crack detection. For example, Zhao et al. [15] used ground penetrating radar to collect multiple time-frequency features to characterize the disease characteristics, and combined with the artificial neural network to realize the intelligent identification of pavement diseases. Ultrasonic testing can also be used to detect cracks in pavements, and Shirahata et al. [16] used creeping waves to monitor fission initiation, which can detect millimeter-scale cracks. Infrared thermal imaging is also used to detect and quantify concrete cracks. Cotič et al. [17] used infrared thermal imaging technology to detect concrete defects and improved the maximum monitoring depth of infrared thermal imaging. However, due to the high cost and difficult operation of inspectors, the above methods can not be widely used in road crack detection. With the rapid development of computer vision and deep learning, image-based defect detection methods have gradually become mainstream. How to improve the recognition and segmentation accuracy of pavement crack images has also become a hot topic around the world. In this section, we summarize the related work within crack detection methods based on deep learning.

In recent years, deep learning methods have been gradually applied to road image segmentation because they can automatically extract target features at multiple scales. Cha et al. [18] proposed a deep convolutional neural network method for crack segmentation, which combines sliding windows to identify crack pixels and their surrounding pixels. However, the inaccuracy of pixel-level labeling may affect the accuracy of the method. Then, Cha et al. [19] proposed a multi-class defect detection method based on Faster R-CNN, which achieved better performance than convolutional neural networks. Long et al. [20] replaced the fully connected layer of the CNN with the convolutional layer and proposed the fully connected network. This method greatly improved the efficiency and accuracy of pixel-level segmentation. Islam et al. [21] used the FCN with an encoder and decoder framework for crack detection, but the coarse feature maps at the top layer of the FCN were not enough to obtain refined segmentation results. Liu et al. [22] first used U-net for concrete crack segmentation. U-net achieves better results on fewer training sets than FCN. Badrinarayanan et al. [23] proposed a new semantic segmentation model SegNet, which consists of an encoder, a decoder, and a pixel classification layer, and achieves better performance than FCN. Zou et al. [24] proposed the DeepCrack model based on SegNet. The larger-scale feature map and more holistic representation enabled the model to detect more crack details. Huyan et al. [25] proposed CrackU-net based on U-net, forming the “U”-shaped model architecture through convolution, pooling, transposed convolution, and concatenation operation. The model differs from U-net by introducing a transposed convolutional layer. The crack detection method proposed by Song et al. [26] obtained crack context information by establishing a multiscale dilated convolution module and introduced an attention mechanism to further refine the high-level features.

## 3. Preliminary

### 3.1. U-net3+

**Figure 1:**
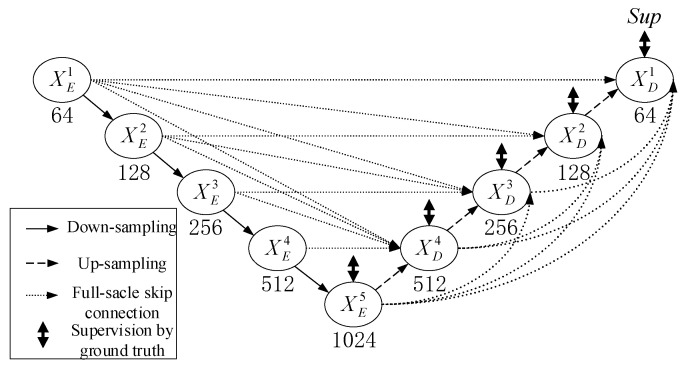
The network architecture of U-net3+.

**Figure 2:**
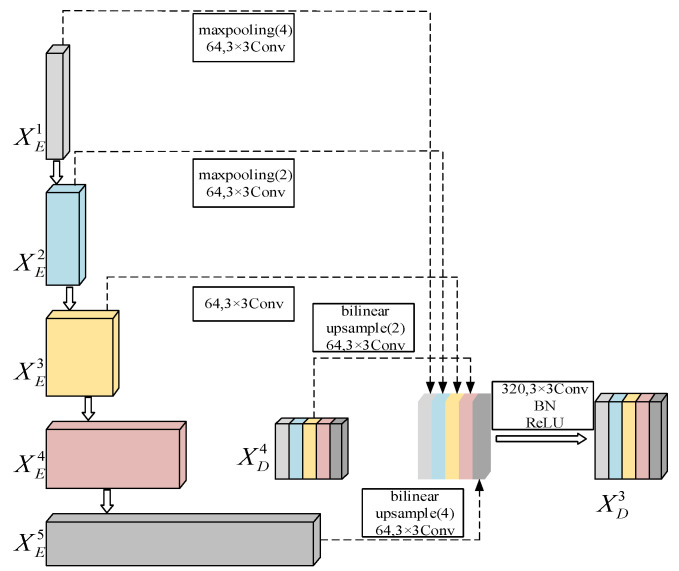
Illustration of how to construct the full-scale aggregated feature map of decoder.

Olaf et al. [27] proposed a U-net network for medical image segmentation. The method has a contracting path that captures context and a symmetric expanding path that enables precise localization. This network can be trained end-to-end using very few images. Huang et al. [28] proposed the U-net3+ network based on the U-net network. Figure 1 depicts the structure of the U-net3+. Research showed that both U-net with ordinary connections and U-net++ with nested and dense connections lack the ability to explore sufficient information from the full scale, so they cannot explicitly learn the locations and boundaries of cracks. The U-net3+ model follows the U-shaped symmetric structure of the U-net. However, each decoder layer of U-net3+ combines smaller- and same-scale feature maps from the encoder and larger-scale feature maps from the decoder. Taking the feature map $X_{D}^{3}$ of the third-layer decoder as an example, Figure 2 illustrates the construction process of the decoder feature map. The feature maps from $X_{E}^{1}$, $X_{E}^{2}$, $X_{E}^{3}$, $X_{D}^{4}$, and $X_{E}^{5}$($X_{D}^{5}$) are combined in order to capture fine-grained details and coarse-grained semantics from full scale. Each decoder stage yields a side output, which is input into the hybrid loss function to supervise and optimize the decoder output.

### 3.2. CGAN

**Figure 3:**
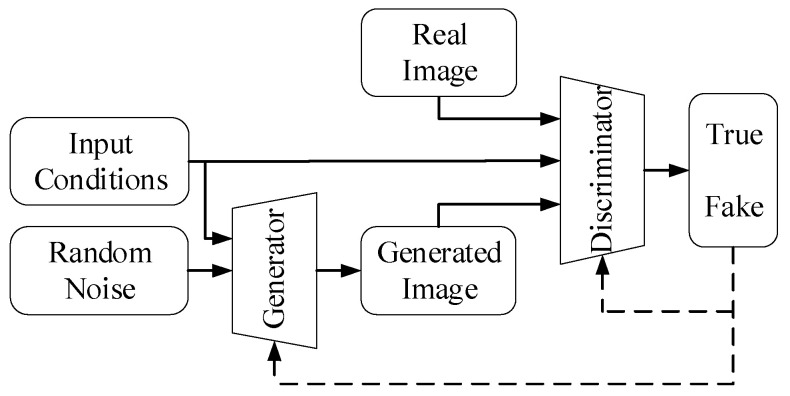
The structure of CGAN.

Goodfellow et al. [29] proposed a generative adversarial network consisting of a generator and a discriminator. The role of the generator is to input random noise and generate an image close to the real image to deceive the discriminator. The discriminator determines whether the input image is from the real world or the generator through repeated game training between the generator and discriminator models until the Nash equilibrium is reached. The generator of GAN can only generate images based on random noise but cannot control the content of the generated images. Therefore, Mirza et al. [30] proposed a conditional generative adversarial network, which added conditions to the input of the generator and discriminator, as shown in Figure 3. It is noteworthy that this network makes the way in which the generator ultimately generates results not completely free and unsupervised but rather generates corresponding results based on a condition.

In the semantic segmentation task, the role of the generator model is to map the source domain image $y\sim p_{sou}$ to the generated segmentation mask $\hat{x}$ and approximate the manual segmentation mask $x\sim p_{seg}$. The generator model can be written as $G(y)=\hat{x}$. Moreover, instead of using an artificially constructed loss function to directly measure the similarity between the generated segmentation mask $\hat{x}$ and manual annotation x, CGAN uses the loss function of the discriminator to judge the segmentation quality of a given source domain image y. The discriminator is a binary classifier. The manual segmentation mask x given the source domain image y is discriminated as true, D(x|y)=1, and the synthetic segmented mask $\hat{x}$ given the source domain image y is discriminated as fake, $D(\hat{x}|y)=0$. The objective function of CGAN could be expressed as a minimax problem:

(1) $$\underset{G}{\min}\;\underset{D}{\max}\;E_{x∼p_{seg},y∼p_{sou}}[\log D(x|y)]+E_{y∼p_{sou}}[\log(1-D(\hat{x}|y))]$$

## 4. Proposed Method

### 4.1. The Generator

**Figure 4:**
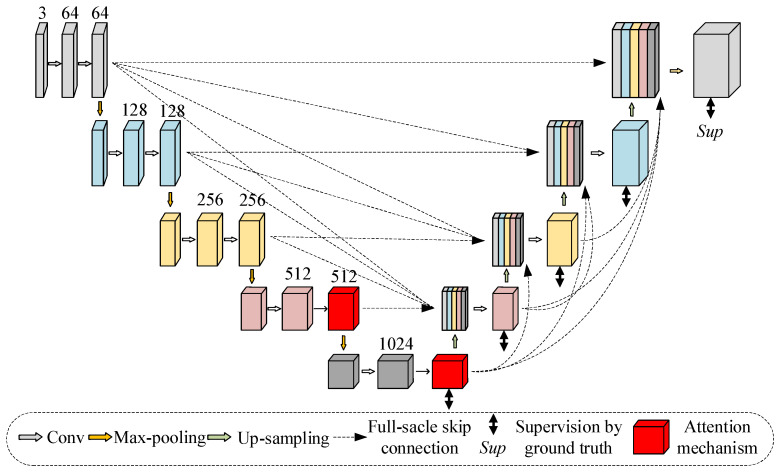
The network architecture of the generator.

In the convolutional neural network, feature maps of different scales describe different feature information of an image. The shallower the network, the smaller the receptive field, and the stronger the ability to represent the geometric details of the extracted shallow features, such as contour, edge, and other information. The deeper the network, the larger the receptive field, and the stronger the semantic representation ability of the extracted deep features, which can determine the attributes of objects. The U-net3+ network uses full-scale skip connections to fuse feature maps of different scales and has a better ability to extract the shallow and deep features of the image. Therefore, we choose U-net3+ as the generator in CGAN. At the same time, in order to solve the inaccurate segmentation problem caused by noise, shadows, and other interference in the image, the attention module is embedded into the U-net3+. The attention module highlights crack features, suppresses noise from the channel and space dimensions, and performs cross-dimensional feature fusion to obtain more complementary crack features so that it improves segmentation accuracy. The overall network architecture of the generator is shown in Figure 4.

**Figure 5:**
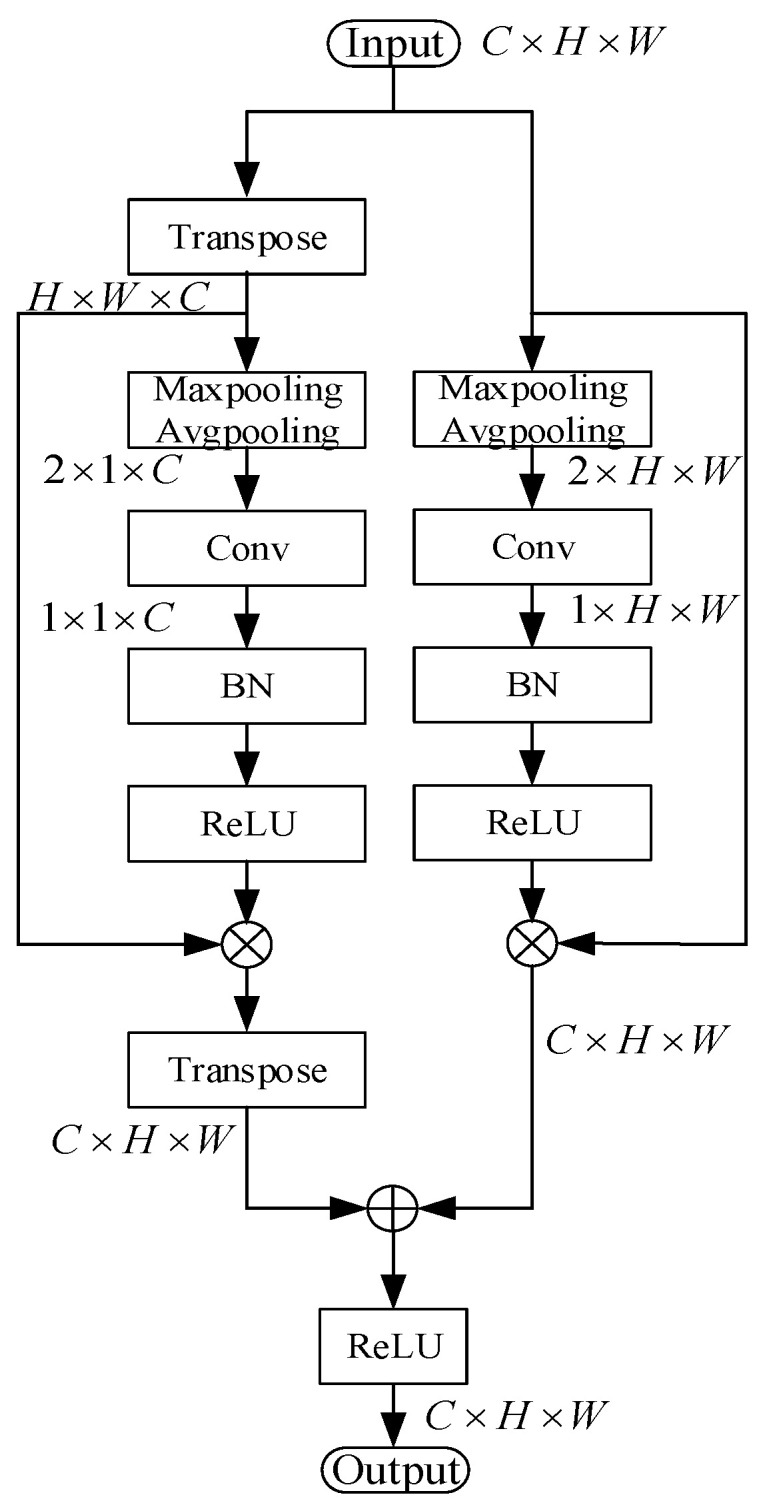
Structure diagram of the parallel attention module.

This paper uses the parallel attention mechanism, and the parallel structure can effectively suppress all kinds of interference, especially the interference of bright noise. As shown in Figure 5, the parallel attention mechanism consists of two parallel branches: The first branch is channel attention, which redistributes the channel weights in the feature map to increase the weights of crack-related channels and reduce the weights of other channels. The second branch is spatial attention, which redistributes the spatial weights more reasonably, gives higher weights to the potential crack regions in the feature map, and reduces the weights of the remaining regions.

1. Channel attention. First, the input feature map is defined as $F\in R^{C\times H\times W}$, and the feature map is transposed to obtain $F^{T}\in R^{H\times W\times C}$. Then, the feature map $F^{T}$ is subjected to maximum pooling and averaging pooling to obtain two 1×1×C feature maps $F_{\max}^{p}$ and $F_{avg}^{p}$. After concatenating the two feature maps, the channel weight map $M_{p}$ is obtained by convolution, batch normalization, and ReLU activation. The following is a mathematical description of $M_{p}$:
  (2) $$M_{p}=\sigma (conv[F_{\max}^{p}⊕F_{avg}^{p}])$$
  where σ denotes the ReLU activation function and ⊕ denotes the concatenate operation.
2. Spatial attention. First, the input feature map is defined as $F\in R^{C\times H\times W}$. Then, the feature map F is subjected to maximum pooling and averaging pooling to obtain two 1×H×W feature maps $F_{\max}^{s}$ and $F_{avg}^{s}$. After concatenating the two feature maps, the spatial weight map $M_{s}$ is obtained by convolution, batch normalization, and ReLU activation. The following is a mathematical description of $M_{s}$:
  (3) $$M_{s}=\sigma (conv[F_{\max}^{s}⊕F_{avg}^{s}])$$
3. Fusion. Finally, the channel weight map $M_{P}$ and the spatial weight map $M_{s}$ are multiplied by the feature map F to obtain two attention feature maps $F_{p}$ and $F_{s}$. The final feature map $G\in R^{C\times H\times W}$ is obtained by adding the two feature maps and using ReLU activation. G can be calculated as follows:
  (4) $$G=\sigma [(M_{p}⊗F)+(M_{s}⊗F)]=\sigma [F_{p}+F_{s}]$$
  where ⊗ denotes the multiplication operation.

### 4.2. The Discriminator

**Figure 6:**
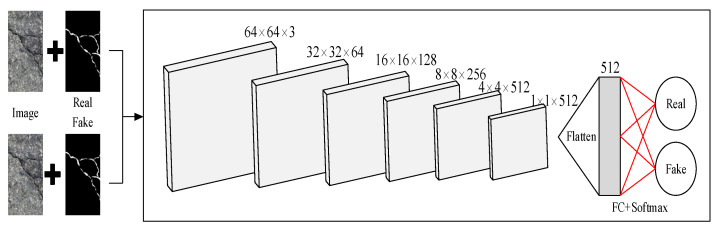
Architecture of the PatchGAN Discriminator.

The discriminator of GAN is a binary classifier to determine whether the input image is from the real world or the generator. We use PatchGAN as the discriminator of CGAN. The original road image is spliced with the manually labeled image and the generated segmented image and then input into the discriminator. The structure of the discriminator is shown in Figure 6. Unlike the traditional discriminator, PatchGAN first divides the image into matrices of size N×N, then judges each matrix to obtain the prediction result according to the average of all the discriminant results. The prediction result is between 0 and 1, which represents the probability of whether the input image is a generated image or a real image.

### 4.3. Design of Loss Function

In order to improve the accuracy of segmented images, we propose a weighted hybrid loss function with two terms. The first is the adversarial loss term, and the second is the segmentation loss term. The weighted hybrid loss function can be written as:

(5) $$𝓁(G,D)=-\lambda \left(E_{x∼p_{seg},y∼p_{sou}}[\log D(x|y)]+E_{y∼p_{sou}}[\log(1-D(\hat{x}|y))]\right)+(1-\lambda )\left({𝓁}_{ce}(\hat{x},x)\right)$$

where G and D represent the generator model and discriminator model, respectively; λ is the weight coefficient; $\hat{x}$ and x represent the segmented mask and manual annotation, respectively; y is the source domain image; D(·) denotes the predicted output of the discriminator.

The training of the CGAN is the process of optimizing the min-max problem of the objective function, which is achieved in practice by alternately training the discriminator and the generator. When training the discriminator, it can be seen from Equation (5) that the discriminator loss is only related to the adversarial loss term, so the loss function can be written as:

(6) $$𝓁(D)=E_{x∼p_{seg},y∼p_{sou}}[\log D(x|y)]+E_{y∼p_{sou}}[\log(1-D(\hat{x}|y))]$$

If only the adversarial loss term is used to train the generator, the output segmentation mask may not always have the same structure as manual annotation [31]. Therefore, adding a segmentation loss term enables the generator to produce the segmented mask that is more similar to manual annotations. The loss function of the generator can be written as:

(7) $$𝓁(G,D)=-\lambda \left(E_{y∼p_{sou}}[\log(1-D(\hat{x}|y))]\right)+(1-\lambda )\left({𝓁}_{ce}(\hat{x},x)\right)$$

(8) $${𝓁}_{ce}(\hat{x},x)=\sum_{i=1}^{H\times W}E_{x_{i},{\hat{x}}_{i}}[-x{\;}_{i}\;log({\hat{x}}_{i})-(1-x_{i})\log(1-{\hat{x}}_{i})]$$

where H×W is the size of the segmentation mask and i is the pixel index of the segmentation mask.

## 5. Experiment and Result Analysis

### 5.1. Experimental Environment and Dataset

The experimental operating platform in this paper is the Ubuntu 18.04 system. The entire algorithm is implemented in the Pytorch framework using the Python3.7 environment. The CPU model is an Intel(R) Xeon(R) E5-2678 v3@2.5GHz, the GPU model is an NVIDIA GeForce RTX 2080 Ti, and the memory of the graphics card is 11 GB.

The dataset used in this paper is the public crack dataset CRACK500. The dataset contains crack images under the influence of various environmental factors such as illumination, shadows, and road obstacles, which can verify whether the proposed method in this paper has a better segmentation ability for crack images with interference. The CRACK500 dataset provides 516 road crack images with a size of 3264×2448. In order to expand the dataset, the images are cropped. Each image is cropped into 16 images of the same size. Then, the images without cracks after cropping are removed. Finally, 3367 cracked images are obtained.

### 5.2. Evaluation Indicators

This paper uses indicators commonly used in semantic segmentation to evaluate the model effect, such as the Dice coefficient, pixel accuracy (Precision), pixel recall (Recall), and F1-score. The expressions of each indicator are as follows:

The Dice coefficient is used to evaluate the similarity between the generated segmented image and the manually labeled image:

(9) $$Dice=\frac{2\left|X\cap Y\right|}{\left|X\right|+\left|Y\right|}$$

where X represents the generated segmentation mask and Y represents the manually annotated mask.

Pixel accuracy represents the percentage of crack pixels detected correctly in detected crack pixels:

(10) $$P_{pixel}=\frac{TP}{TP+FP}$$

where TP represents the number of Correct detection pixels belonging to the crack region, and FP represents the number of False detection pixels belonging to the crack region.

Pixel recall represents the proportion of correctly detected crack pixels to all crack pixels:

(11) $$R_{pixel}=\frac{TP}{TP+FN}$$

where FN represents the number of False detection pixels belonging to the background region.

F1-score is used to measure the accuracy of the model:

(12) $$F_{1}=\frac{2P_{pixel}R_{pixel}}{P_{pixel}+R_{pixel}}$$

### 5.3. Experimental Results

The CGAN network consists of two parts: the generator and the discriminator. The distribution training strategy is adopted during training: (1) The road crack image is spliced with the manually labeled image. Then, it is input into the discriminator network, using the real sample to train the discriminator. (2) Keeping the parameters of the generator model fixed, the segmented image generated by the generator is spliced with the road crack image to train the discriminator. (3) Keeping the parameters of the discriminator model fixed, the generated segmented image is spliced with the road crack image. It is inputted into the discriminator to obtain the adversarial loss, and the generator is trained by the adversarial loss. (4) Repeat steps 1–3. The training of the network is completed after 15,000 iterations. During training, we set the batch size to 4, the initial learning rate to 0.01, and the decay rate of the learning rate to 0.1. The learning rate is updated by tracking the loss, and the learning rate is reduced when the loss does not decrease after 500 iterations.

**Figure 7:**
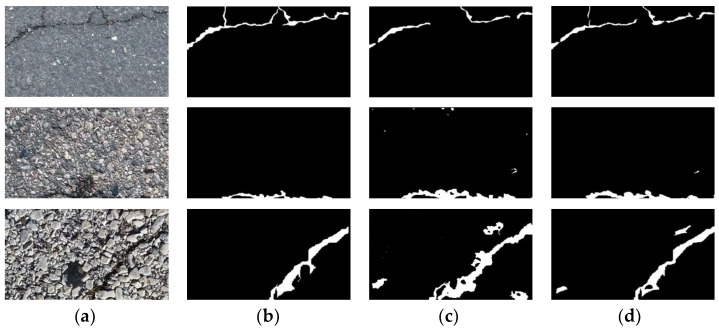
Visualization results of different attention mechanisms. (**a**) Road Image; (**b**) Ground Truth; (**c**) CBAM; (**d**) Ours.

In order to verify the performance of the parallel attention module, this paper selects four widely used attention modules: SENet, CBAM, SAM, and ECA-Net, and conducts a comparative experiment with the parallel attention module proposed in this paper. The comparison results are shown in Table 1. In this experiment, U-net3+ is selected as the benchmark network. It can be seen from Table 1 that after adding the attention modules SENet and SAM, the pixel accuracy of the network increases by 1% and 1.3%, respectively, compared with the benchmark network. However, the pixel recall decreases by 1.1% and 1.4%, respectively, resulting in a decline in the overall performance of the model. The results of CBAM and the proposed attention are better than that of the benchmark network, and the overall performance of the model increases by 0.7% and 1.2%, respectively, because both channel attention and spatial attention are added. CBAM is a serial structure that cannot effectively suppress bright interference and performs poorly in road crack segmentation tasks. In order to verify the advantages of parallel attention, some images in the test set are selected to compare the attention of CBAM and this paper visually. The results are shown in Figure 7.

When the cracks in the image are thin and the background interference is small, as shown in the first row of Figure 7, the road crack image segmented by the CBAM attention mechanism is obviously missing. Although the attention mechanism proposed in this paper also has the phenomenon of missed detection, the crack structure is relatively whole. Both attention mechanisms can segment the crack relatively completely when the image crack thickness is moderate and the background interference is small, as shown in the second row of Figure 7. When the cracks in the image are thick and the background has large interference, as shown in the third row of Figure 7, the CBAM attention mechanism will falsely detect a lot of interference information as cracks, and the crack information segmented by the attention mechanism proposed in this paper is more accurate, with detailed information. Therefore, the parallel attention mechanism proposed in this paper can effectively suppress various interferences and improve detection performance.

**Figure 8:**
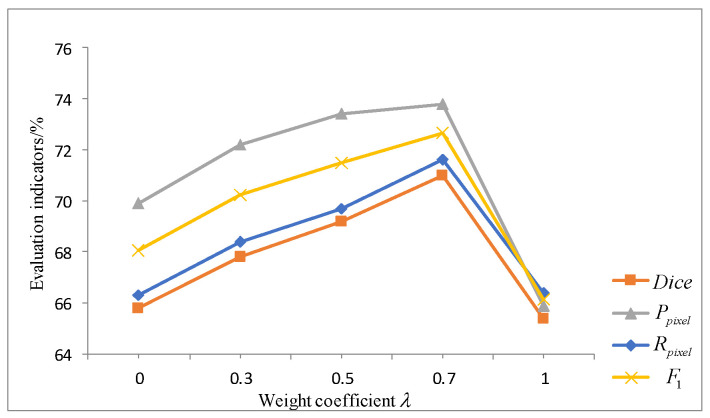
Experimental results of hybrid loss functions with different weight coefficients.

In order to verify the effectiveness of the hybrid loss function proposed in this paper on the road crack segmentation task and determine the optimal value of the weight coefficient x, x is set to 0, 0.3, 0.5, 0.7, and 1 to train the network. Figure 8 shows the effect of the loss function on model performance with different weight coefficients x. It can be seen from Figure 7 that the indicators of the model fluctuate under different values. When x is 0, the U-net3+ network is only trained using the cross-entropy loss. When x is 1, i.e., only adversarial loss is used for training, the performance of the model is worst. When x is 0.7, the overall indicators of the model are optimal. Therefore, the weight coefficient of the loss function is set to 0.7 when the model is used.

In the segmentation task of binary classification, in addition to the binary cross entropy (BCE) loss function we use, the loss functions often used are Dice loss, IoU loss, and so on. In order to study the influence of different loss functions on network performance, we choose Dice loss and IoU loss to perform comparative experiments with the same weight coefficient. The experimental results are shown in Table 2.

Dice loss is a set similarity measure function, usually used to calculate the similarity of two samples, which can be written as:

(13) $${𝓁}_{Dice}(\hat{x},x)=1-\frac{2\times \sum_{i=1}^{H}\sum_{j=1}^{W}\hat{x}(i,j)x(i,j)}{\sum_{i=1}^{H}\sum_{j=1}^{W}\left[\hat{x}(i,j)+x(i,j)\right]}$$

where $\hat{x}$ and x represent the segmented mask and manual annotation, respectively; H×W is the size of the segmentation mask.

The IoU loss is more focused on small targets and is more inclined to mine the foreground area during training, which can be written as:

(14) $${𝓁}_{Dice}(\hat{x},x)=1-\frac{\sum_{i=1}^{H}\sum_{j=1}^{W}\hat{x}(i,j)x(i,j)}{\sum_{i=1}^{H}\sum_{j=1}^{W}\left[\hat{x}(i,j)+x(i,j)-\hat{x}(i,j)x(i,j)\right]}$$

As can be seen from Table 2, the pixel recall of Bce loss is smallest, because the BCE loss function is more inclined to identify the crack background than the crack, so even though the pixel accuracy is high, the recall is still low. Although the recall rate of Dice loss and IoU loss is higher than that of Bce loss, the pixel accuracy is lower and there are large errors in crack segmentation. When Dice loss and IoU loss are used as loss functions, there is little difference in network performance because both are measured by the ensemble similarity. From the results, it can be seen that adding the binary cross entropy loss function to the adversarial loss is more effective than the other two loss functions, which is due to the advantage of Bce loss in the dense pixel classification task. However, we do not explore the effect of combining multiple segmentation losses and adversarial losses, which is also a research direction to continue to improve network performance.

**Figure 9:**
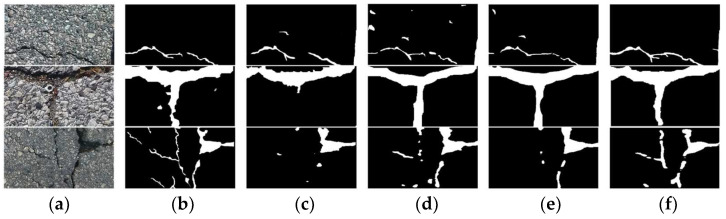
Visualization results of different networks. (**a**) Road Image; (**b**) Ground Truth; (**c**) U-Net; (**d**) U-Net3+; (**e**) U-Net+CGAN; (**f**) Ours.

In order to verify the superiority of CGAN in the task of road crack segmentation, we use the Crack500 dataset to train U-Net, U-net3+, and U-net+CGAN networks and compare them with the proposed model. The result of the comparative experiment is shown in Table 3. When training the U-net+GAN network, the loss function adopts the hybrid loss function with a weight coefficient of 0.7. It can be seen from Table 3 that the evaluation indicators of U-Net+CGAN are higher than those of U-Net, and the evaluation indicators of U-Net3++CGAN are higher than those of U-Net3+, indicating that the combination of the original segmentation network and CGAN is beneficial to the segmentation task. The Dice and F1 of the proposed method are best, which are 71% and 72.7%, respectively, and are increased by 10.7% and 9.9%, respectively, on the basis of U-net. In order to more intuitively reflect the segmentation differences between different networks, some of the test results are selected for visual analysis, as shown in Figure 9. The first and second columns are the original and manually labeled images, respectively. The remaining columns are the segmentation results of U-Net, U-Net3+, U-Net+CGAN, and the proposed method. It can be seen from Figure 9 that U-net3+ has a stronger segmentation ability than U-net. After adding CGAN, the anti-interference ability of the original network is improved.

In order to verify the superiority of the proposed method in the road crack segmentation problem, we compare the proposed method with several other popular segmentation methods: DeepCrack [11], SegNet [23], and Fast-SCNN [32]. Among them, DeepCrack is composed of an extended fully convolutional network and a deeply supervised net, which can effectively learn the hierarchical features of multi-scenes and multi-scale fractures, and use guided filtering for post-processing to make the detection results more accurate. SegNet consists of an encoder network, a corresponding decoder network, and a pixel classification layer. The encoder structure is the same as the convolutional layer in VGG16, and the decoder uses upsampling when mapping low-resolution features to input resolution, significantly reducing the number of parameters. Building on the two-branch method for fast segmentation, Fast-SCNN introduces a “learning to downsample” module, which computes low-level features for multiple resolution branches at the same time. Each network model is trained under the same training dataset and training conditions. The comparison results are shown in Table 4. In Table 4, the speed of the fracture detection model is measured in frames per second (FPS). As can be seen from Table 4, the crack segmentation performance of the proposed method is greatly improved compared with other methods. Compared with DeepCrack, SegNet, and Fast-SCNN, the F1 score of the proposed method is improved by 9.9%, 5.8%, and 5.3%, respectively. In terms of detection speed, the FAST-SCNN method is nearly twice as fast as the method in this paper, because its network structure does not use the encoder–decoder structure, but adopts the double-branch structure, and the depth-separable convolution is used to further reduce the number of parameters and computations. Although U-net3+ reduces the number of parameters and improves the detection speed on the basis of U-net, its real-time performance still needs to be further strengthened. In the future, model pruning or model distillation can be used to further reduce the number of parameters and improve the speed of the network.

**Figure 10:**
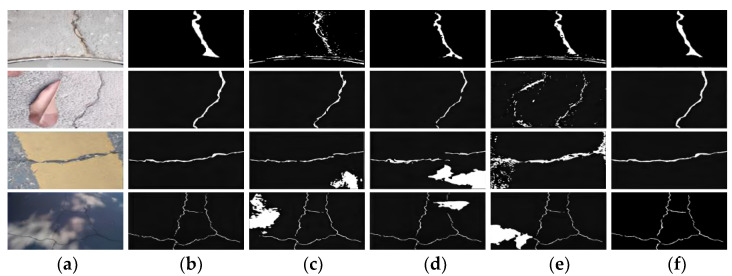
Visual comparison results of different algorithms in crack images with noise. (**a**) Road Image with Noise; (**b**) Ground Truth; (**c**) DeepCrack; (**d**) SegNet; (**e**) Fast-SCNN; (**f**) Ours.

In order to show the superiority of the proposed method more intuitively, we select some test set images for visual segmentation comparison with other crack segmentation methods. The selected images contain complex disturbances such as manhole covers, fallen leaves, driveway lines, and shadows. The comparison results are shown in Figure 10. It can be seen from Figure 10 that under complex background conditions, other algorithms have weak anti-interference to noise images, and the expression of crack boundary information is not accurate enough. The false detection rate of the proposed method is relatively small, which indicates that the parallel attention mechanism improves the attention of the model to the fracture characteristics and suppresses the irrelevant interference. In addition, the integrity and continuity of the cracks segmented by this method are better, which shows that the introduction of CGAN improves the attention to the high-order consistency of data and obtains more accurate and smoother segmentation results.

**Figure 11:**
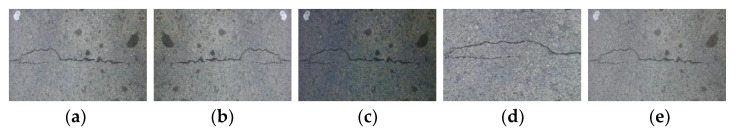
Different types of data augmentation methods. (**a**) Original Image; (**b**) Flipping Transformation; (**c**) Contrast Transformation; (**d**) Random Cropping; (**e**) Noise.

Data augmentation is a method of transforming the training set image in the training stage to improve the generalization ability of the model. At present, because there are few pavement crack images and the workload of labeling cracks at the pixel level in the image is large, the amount of data in the public dataset is small. The segmentation model trained directly on the dataset is prone to overfitting and has a poor generalization ability. Therefore, the method of data enhancement is usually used in the task of pavement crack segmentation. Because the crack image has the characteristics of variable crack direction, low brightness, and various noise, the data enhancement methods we choose include flipping transformation, contrast transformation, random cropping, and noise interference. As shown in Figure 11, all the data augmentation methods are as follows. To explore the performance improvement of the proposed method by data enhancement, we train the network using the enhanced dataset and compare its performance with that of the network without data augmentation. The comparison results are shown in Table 5.

As can be seen from Table 5, the data augmentation method can indeed improve the performance of the proposed method, with the pixel accuracy increased by 1.7%, the pixel regression rate increased by 2%, and the overall segmentation performance increased by 1.8%. It is worth mentioning that the performance improvement brought by data augmentation only increases the training time in the training stage, and does not increase the computational overhead in the inference stage.

## 6. Conclusions

We studied the problem of pavement crack segmentation in the process of road maintenance, i.e., crack segmentation is inaccurate due to complex backgrounds and various disturbances of the pavement. Therefore, we proposed a pavement crack segmentation algorithm based on CGAN to accomplish this task. The purpose of introducing CGAN into the crack segmentation task is to evaluate the crack pixels as a whole, to obtain more accurate and smooth segmentation results. The generator adopted the U-Net3+ network and introduced the parallel attention module in U-Net3+. The parallel fusion of channel attention and spatial attention can highlight crack features and suppress interference features to obtain more complementary crack features. The discriminator adopted the PatchGAN structure. In addition, a weighted hybrid loss function was used to exploit the pixel difference between the segmented image and the annotated image to improve the segmentation accuracy further. The dataset used in the experiment was Crack500. First, in order to verify the effectiveness of the proposed parallel attention, we performed an ablation experiment of the attention module. The comparison results with the benchmark model and other attention mechanisms showed that the proposed parallel attention module can effectively eliminate interference and the visual comparison result was shown. Then, in order to verify the effectiveness of the hybrid loss function and find the best weight coefficient, we selected the hybrid loss function with different weight coefficients to train the network. To explore the influence of different segmentation losses on the mixing function, a comparative experiment was performed using different segmentation loss functions. Third, for verifying the superiority of the introduction of CGAN, we conducted ablation experiments. The experimental results showed that the performance of the segmentation model is improved after the introduction of CGAN. Finally, the comparison test with other crack segmentation models showed that the proposed method could achieve superior performance over existing methods.

## Figures and Tables

**Table 1:** The results of different attention mechanisms.

Method	$P_{pixel}/\%$	$R_{pixel}/\%$	$F_{1}/\%$
U-Net3+	69.9	66.3	68.0
+SENet	70.9	65.2	67.9
+CBAM	71.0	66.6	68.7
+SAM	71.2	64.9	67.9
+ECA-Net	73.1	63.9	68.2
+Ours	71.1	67.5	69.2

**Table 2:** The results of different loss functions.

Loss Function	$P_{pixel}/\%$	$R_{pixel}/\%$	$F_{1}/\%$
Adv+BCE	73.8	71.6	72.7
Adv+Dice	71.4	73.6	72.5
Adv+IoU	71.3	73.4	72.3

**Table 3:** The results of different networks.

Network	Dice/%	$P_{pixel}/\%$	$R_{pixel}/\%$	$F_{1}/\%$
U-Net	60.3	65.2	60.6	62.8
U-Net3+	65.8	69.9	66.3	68.0
U-Net+CGAN	63.8	67.8	63.8	65.7
U-Net3++CGAN	71.0	73.8	71.6	72.7

**Table 4:** Performance comparison of different crack detection algorithms.

Algorithm	$P_{pixel}/\%$	$R_{pixel}/\%$	$F_{1}/\%$	FPS	Params/M
DeepCrack	61.2	64.4	62.8	25.6	30.9
SegNet	67.2	66.6	66.9	18.7	16.3
Fast-SCNN	65.5	69.6	67.4	67.5	1.2
Ours	73.8	71.6	72.7	34.8	29.0

**Table 5:** The comparison results of data augmentation.

Data Augmentation	$P_{pixel}/\%$	$R_{pixel}/\%$	$F_{1}/\%$
	73.8	71.6	72.7
√	75.5	73.6	74.5

## Data Availability

Data sharing is not applicable.
